# Moving from development to implementation of digital innovations within the NHS: myHealthE, a remote monitoring system for tracking patient outcomes in child and adolescent mental health services

**DOI:** 10.1177/20552076231211551

**Published:** 2023-11-09

**Authors:** Anna C Morris, Zina Ibrahim, Omer S Moghraby, Argyris Stringaris, Ian M Grant, Lukasz Zalewski, Stuart McClellan, Garry Moriarty, Emily Simonoff, Richard JB Dobson, Johnny Downs

**Affiliations:** 1 4958 South London and Maudsley NHS Foundation Trust, London, UK; 2 Department of Child and Adolescent Psychiatry, Institute of Psychiatry, Psychology and Neuroscience, 4616 King's College London, London, UK; 3 Department of Biostatistics and Health Informatics, Institute of Psychiatry, Psychology and Neuroscience, King's College London, London, UK; 4 Emotion & Development Branch, 25944 National Institute of Mental Health, National Institutes of Health, Bethesda, MD, USA; 5 130346 NIHR South London and Maudsley Biomedical Research Centre, London, UK; 6 Health Data Research UK London, University College London, London, UK; 7 Institute of Health Informatics, University College London, London, UK; 8 NIHR Biomedical Research Centre, University College London Hospitals NHS Foundation Trust, London, UK

**Keywords:** Digital health, general, health, general, online, paediatrics, medicine, electronic, personalised medicine, mHealth, psychology, mental health, wellbeing, mixed methods, studies, outcomes

## Abstract

**Objective:**

This paper aims to report our experience of developing, implementing, and evaluating myHealthE (MHE), a digital innovation for Child and Adolescents Mental Health Services (CAMHS), which automates the remote collection and reporting of Patient-Reported Outcome Measures (PROMs) into National Health Services (NHS) electronic healthcare records.

**Methods:**

We describe the logistical and governance issues encountered in developing the MHE interface with patient-identifiable information, and the steps taken to overcome these development barriers. We describe the application's architecture and hosting environment to enable its operability within the NHS, as well as the capabilities needed within the technical team to bridge the gap between academic development and NHS operational teams.

**Results:**

We present evidence on the feasibility and acceptability of this system within clinical services and the process of iterative development, highlighting additional functions that were incorporated to increase system utility.

**Conclusion:**

This article provides a framework with which to plan, develop, and implement automated PROM collection from remote devices back to NHS infrastructure. The challenges and solutions described in this paper will be pertinent to other digital health innovation researchers aspiring to deploy interoperable systems within NHS clinical systems.

## Background

Patient-Reported Outcome Measures (PROMs) are recognised as indispensable clinical tools to document and enhance patient experiences,^1^ and support outcome improvement.^1–4^ The healthcare policy in the United Kingdom recommends routine outcome monitoring in mental health services for children and young people to measure treatment quality and utility.^5–7^ Structured reports from families, including children and their caregivers, provide clinicians with information to assess the efficacy of treatment regimens for their patients, encourage more transparent and collaborative patient–clinician communication, and enhance therapeutic alliance.^8–11^ Evidence from child mental health research suggests that symptoms improve faster in patients of clinicians who received PROMs feedback during treatment compared to patients of clinicians who received less or no feedback from standardised measures.^3,12,13^ However, long-term PROM collection is not part of routine practice in mental health clinics^14,15^ and Child and Adolescent Mental Health Services (CAMHS). Survey findings suggest that the guidelines for recording PROMs are adhered to in as few as 6%–30% of clinical cases.^16–18^ Furthermore, a study analysing the health records of child and adolescent patients diagnosed with ADHD at the South London and Maudsley National Health Service Foundation Trust (SLaM NHS FT), one of the largest mental health institutes in Europe, found that less than 1% of longitudinal PROMs are recorded.^19^

Low rates of PROM recording, especially in CAMHS,^20^ have been attributed to patient and service level factors.^17,21^ Despite the known value of PROMS, demanding workloads and limited time and resources mean that clinical teams are often too stretched to systematically administer and collect these questionnaires, tending to rely on clinical experience to record patients’ progress.^22^ Low response rates from families to structured outcome measures and lack of feedback available to both clinicians and patients are contributing factors.^23^ In response, online electronic PROM (ePROM) systems have been designed and provisioned to support outcome monitoring in healthcare settings by offering a practical solution to identified barriers to collecting paper-based PROMs.^24,25^ Moreover, considering the increased number of patients accessing healthcare online due to the emergent COVID-19 pandemic, enthusiasm for web-based monitoring portals is growing to facilitate e-form collection virtually.^26^ In the context of paediatric outpatient services, evidence suggests that both patients and their caregivers favour electronic rather than pen-and-paper measure completion when allowed to complete either.^27^ Despite the promise of technology to transform the way that the National Health Services (NHS) collects stores and displays clinical information,^28–30^ digitalisation in these areas is reported to be around 20 years behind private-sector healthcare providers in the UK.^31^ This suggests that cultural factors within the NHS and the mechanics of developing e-platforms may disproportionately affect the organisation's efforts to improve healthcare by introducing paperless strategies. This has led to recent calls from NHS England for digital reforms across all services provided within the system, emphasising the importance of digital infrastructure interoperability.^32^

A plethora of ePROM systems now exist that target the clinical management of both physical and behavioural health,^33–37^ though the success of tools provisioned in mental health settings is more varied.^38–40^ Furthermore, the benefits of remote monitoring platforms may be limited by their functionality. For example, while a recent review of ePROM collection tools identified 33 exemplars in the field of clinical oncology, only 37% of these allowed respondents to complete PROMs outside the clinical setting, i.e. from their own home, and just 44% of remote monitoring tools could integrate collected data directly to patient's electronic health records (EHRs).^41^ Moreover, electronic platforms often rely on input from clinicians to set the type and frequency of follow-up outcome measures required,^41^ thus limiting the potential time and effort savings projected from ePROM delivery.^42^ At present, the majority of ePROM systems are developed as standalone platforms, independent of the EHRs responsible for handling routinely collected health information, therefore, patient consent is normally required before ePROM systems can be used to request clinical information at the patient level.^43^ As such, the integration of EHR-embedded platforms could substantially reduce the burden associated with the collection of routine outcome measurements, without the need for patient consent.

In this paper, we discuss myHealthE (MHE), which is to the best of our knowledge the first documented web-based outcome monitoring system that specifically addresses the challenges associated with low return rates of primary carer outcome measures in UK CAMHS, through direct integration with patients’ EHRs. Notably, MHE automates the scheduling, delivery, and collection of electronic parent-reported questionnaires at pre-defined post-treatment intervals, i.e. 6 months after baseline completion, as per national guidelines for PROM collection in child mental health services.^5^ The system further enables reporting of collected measures through both patient's EHRs and a patient-facing platform to support measurement-based care and service evaluation. In describing the application, we aim to accomplish three main objectives. Firstly, we provide a reflective account of our experience in developing, deploying, testing, and evaluating MHE, in relation to pen-and-pencil-based questionnaire uptake. Secondly, we offer a comprehensive description of the resulting system, covering its technical architecture, data flow, and the limited human input required for functionality. This is done by describing the interdisciplinary approach required to build a secure cloud-based environment capable of hosting MHE, to permit the secure processing and storage of clinical data, aligned with key information governance and information technology (IT) clearance processes. We propose potential facilitators to help to support and streamline operability for other future digital initiatives in the NHS, as well as provide clarity around issues of product ownership and maintenance responsibilities. Thirdly, we discuss the key barriers we encountered during the implementation of MHE into clinical practice to bridge the gap between frontline care and innovation research. Here, we provide an implementation strategy, guided by a well-established conceptual framework, the Consolidation Framework for Implementation Research (CFIR),^44^ which helped to ensure that clinical service support was developed and maintained throughout our implementation efforts.

## Methods

### myHealthE team

The interdisciplinary system development team was led by a King's Health Partnership group combining academic and clinical expertise from King's College London (KCL) and SLaM NHS FT. A university-based research team, comprising a clinical academic lead (JD) and research assistant (AM), was responsible for daily project management and implementation and worked closely with KCL-employed health informaticians (ZI, IG, LZ) and SLaM NHS FT information governance, digital clinical systems leads (SM, GM) to prepare MHE for clinical use.

### myHealthE overview

MHE enables repeat, automated patient progress tracking via the collection of the caregiver-reported Strengths and Difficulties Questionnaire (SDQ-P), a brief 25-item measure of emotional and behavioural childhood psychopathology.^45^ This questionnaire has been validated for use in clinical settings and randomised controlled trials^46^ and is authorised for use in SLaM through a sub-license granted by the NHS Digital Copyright Licensing Service. Specifically, MHE requests SDQ-P reports from families who are due to provide additional questionnaire information 6 months after completing their first measure at the beginning of treatment, as per clinical guidance.^5^ To achieve this, MHE integrates two primary components: firstly, a multi-agent tracking programme, adapted from the previously described APPROaCh (Agent Platform for automating patient PROvided Clinical outcome feedback) framework,^47^ comprising the agents DataManager, UnitManager, and PatientFollowUp – and an associated database. Together, these agents enable MHE to monitor internal patient health records, import patients’ existing baseline questionnaire responses, enrol them in the system, and create a patient-specific schedule for notifying the caregiver to complete follow-up measures, entirely independent of human input. Secondly, a patient-facing web application is used, which allows the caregiver to log in and fill out online questionnaire forms to log new measures on a schedule that is pre-determined by the tracking agent and visualise the results of their questionnaires. These additional questionnaire results are logged into the same database that the tracking agent interfaces with, creating a feedback loop aligned to the patient-specific schedule originally determined by the tracking agent.

### myHealthE development

#### Establishing system specifications

Initial specification requirements were developed by the MHE team and focused on three main themes: user experience, security, and scalability. Early examples of MHE specification requirements included the need to identify a (cloud-based) server suitable for hosting the application and patient-identifiable information (PII), a reliable and secure text and email communication mechanism, and a safe method of caregiver registration verification. Having developed MHE from an NHS governance and engineering perspective, we contracted a local (South London) digital commercial supplier specialising in website and application development to help design an engaging and user-friendly patient interface following thorough KCL procurement procedures.

#### Proposed system data flow and technical components build

**Figure 1.**
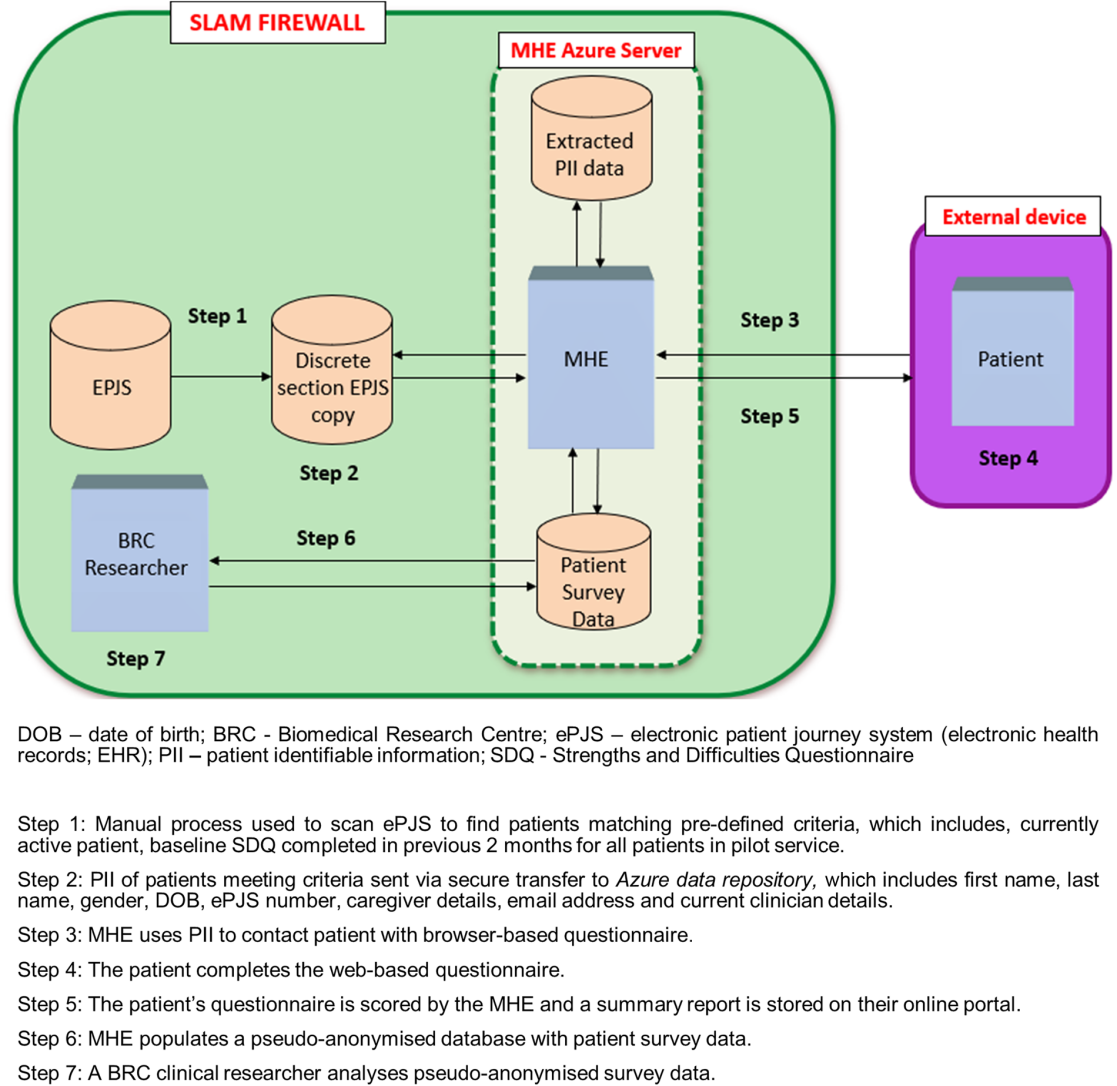
MHE extraction and imputation data flow process.

Figure 1 illustrates the MHE extraction and imputation data flow process. To align with hospital standards for collecting and managing patient-identifiable information, we deployed the system on a virtual server that was provisioned within SLaM's Microsoft Azure cloud subscription; this provides direct, private integration with SLaM's IT infrastructure (including the EHR), protected by the same institutional firewalls and relevant cybersecurity protocols as the rest of the SLaM digital estate.

The prescribed SLaM server hosts two distinct databases, which are populated via the MHE agent framework. Firstly, a SQL patient identifier database is populated through an MHE-specific script (implemented by the SLaM Systems Team) in SLaM's EHR (also known as the electronic Patient Journey System; ePJS). The script extracts child and caregiver PII; these include first name, last name, sex, date of birth, and contact details (e.g. mobile numbers and email addresses) for specific patient populations. The selected children include active CAMHS patients who require a follow-up parent-reported SDQ-P^47^ to be completed (patients who have a completed baseline SDQ-P with no follow-up SDQ-P entered). Secondly, a dataset populated with patient survey data that is generated through patient caregivers completing online SDQ-P measures. This dataset is identifiable via a unique MHE-generated identification number, allocated to each child enrolled on the system, accessible only to the MHE research team. MHE also provides a mechanism for SLaM to know which caregivers are happy to be contacted about the prospect of enrolling in future research studies being conducted at SLaM.

#### Anticipated translational challenges

After determining prerequisite application architecture and data flows, the interdisciplinary group identified several potential barriers which could restrict the development and transfer of MHE from a research environment into clinical practice.

1. *Legacy systems:* Electronic Healthcare Records and Digital Systems (especially in the UK) have largely comprised specialised proprietary technologies. As with many NHS Healthcare providers, SLaM EHR software systems were not designed to be interoperable, nor remotely accessible, but built for meeting the immediate clinical and clerical needs of recording and storing captured data from patient interactions. Building innovations that can enhance the capabilities of these proprietary systems within the NHS has been hampered by the lack of centrally agreed data standards for sharing, or open software systems for shared development. With no mandate for interoperability, it has been historically difficult for NHS organisations to encourage EHR and technology suppliers to adopt Digital Health Monitoring Systems (DHMS) from research into clinical practice.^48^
2. *Introduction of cloud-based solutions:* Recent guidelines published by NHS Digital^48^ recommend that all NHS services transition from using locally managed servers to store patient information to public cloud-based solutions. This recommendation releases NHS organisations from some of their maintenance obligations to continually improve and upgrade local systems and permits the development of digital products that are not constrained by data storage or processing limits. Because of the recent change in guidance, most NHS organisations are yet to establish their in-house standards and processes for developing DHMS cloud-based environments suitable for the safe processing and storage of clinical data.
3. *Data security and information governance:* MHE was developed to replace a pen-and-paper method for collecting sensitive and personal clinical data. The security parameters for our application needed to provide full system protection, in line with SLaM's policy for existing electronically held patient records. New relationships would need to be established between KCL and SLaM's governance and IT personnel, to enable clear communication throughout the project phases to conduct testing, evaluation, and deployment of MHE as a digital innovation.
4. *Resource barriers:* At an organisational and system-wide level, the NHS is not financially structured to support innovation design, delivery, or dissemination in addition to existing workload.^49^ We expected to encounter project delays during the development stage due to a lack of designated organisational personnel to oversee project management and the construction of innovative digital technologies.^50,51^
5. *Product ownership:* MHE was created in a university research and development environment as a system to enhance routine clinical practice. The business case had yet to be fully established for SLaM to adopt this innovation to be maintained as business-as-usual, as with all other clinical systems. Hence, MHE would require support and resources from already stretched SLaM services, in addition to their existing workloads.
6. *Lack of engagement from the clinical team:* Enthusiasm within the clinical service selected for MHE implementation could diminish if protracted technical delays were encountered over the course of the deployment.

#### Implementation setting

MHE implementation took place at Kaleidoscope, a community paediatric and children's and young people's mental health centre based in Lewisham, South London, within the Lewisham Neuro-developmental Team (NDT). The team consisted of a clinical lead consultant child psychiatrist, an NDT service manager, two senior supervisory clinical psychologists, three clinical psychologists, and two family therapists. Implementation efforts began in August 2017, culminating in a 12-week single-blinded parallel group feasibility pilot randomised control trial conducted between the 11 February 2019 and 14 May 2019. This trial aimed to examine MHE-facilitated SDQ-P completion rates for children receiving care from the NDT. Full details of the trial rationale, aims, methods, and statistical analysis are available here (http://doi.org/10.1111/camh.12571).

#### Implementation strategy

Too often, innovations in digital health fail to reach frontline care owing to poorly defined implementation strategies. Adoption of electronic systems into established clinical settings is a complex process, reliant on several important multi-level innovation and organisational factors.^52^ As such, we use the CFIR framework^44^ to pre-determine variables that might impact the success of MHE implementation. Briefly, the CFIR describes five key domains to be considered during implementation: (a) intervention characteristics, (b) the outer setting, and (c) the inner setting in which implementation occurs, (d) characteristics of persons participating in the implementation, and (e) the implementation process itself. The framework is intended to be used flexibly by the specific context in which implementation is taking place. In this application, informed by other researchers’ experience of implementing health informatic systems,^3,38^ we anticipate barriers mostly relating to intervention characteristics. Specifically, these barriers related to the relative advantage of MHE compared to routine data collection, local adaptability, and MHE complexity. Possible inner settings difficulties identified included implementation climate and readiness for implementation as well as individual characteristics, namely, clinicians’ knowledge and beliefs. Accordingly, we aim for MHE implementation to follow a four-staged implementation approach that is expected to take 6 months to complete. The staged approach is intentionally designed to alleviate any intervention concerns and foster local ownership of the system.

1. *Baseline evaluation:* To provide a baseline assessment of caregiver-reported SDQ completion rates for SLaM CAMHS, we will run searches through Clinical Records Interactive Search (CRIS), a database comprised of de-identified records for all patients accessing SLaM services.^53–55^ SLaM provides community outpatient CAMHS and specialist services to a catchment area of 1.3 million residents across four boroughs in South London, offering additional national and specialist services to children and young people across the UK. The socioeconomic characteristics of the SLaM catchment area are some of the most diverse in the country, including boroughs with a higher percentage of Afro-Caribbean residents compared to the rest of England and Wales (https://www.ethnicity-facts-figures.service.gov.uk), as well as the highest proportion of children experiencing income deprivation (https://www.gov.uk/government/statistics/english-indices-of-deprivation-2015). The CRIS data resource has received overarching research ethics approval for secondary data analysis, and therefore, individual patient written consent was not required for this study (Oxford REC C, reference 18/SC/0372). This analysis will objectively capture evidence of the unmet clinical need for follow-up outcome measure collection in CAMHS and can be used to promote the potential benefit of using an automated electronic PROM collection tool relative to more labour-intensive paper methods within the clinical implementation setting. This will allow us to assess whether our automated electronic system outperforms current SLaM SDQ-P data collection procedures.
2. *Orientation:* This stage will focus on on-site preparation, achieved through regular email correspondence and face-to-face meetings with NDT clinical lead and service manager, as well as staff consultations at clinical team meetings. This practice will be used to discuss the aims and expectations of the project, assess the service's current local capacity and readiness for MHE adoption, and develop fundamental communication channels within the service. Information obtained at this stage will be used to circumvent potential inner setting concerns, for example, discussing how MHE will be incorporated into existing workflows, demonstrating managerial support for MHE implementation, and ensuring that team members perceive themselves as equally valued, and knowledgeable contributors in the change process.^44^
3. *Stakeholder engagement:* Stakeholders will be familiarised with MHE functionality and allowed to provide system feedback through multiple separate staff and parent group interactive demonstrations of a prototype MHE system. These sessions provided stakeholders with the opportunity to voice opinions about the characteristics of the system and suggest desired system alterations.
4. *System refinement:* Understanding that the application meets stakeholders’ needs is imperative to the success of the application. Therefore, feedback from the stakeholder engagement stage will be used to iteratively refine the MHE prototype ready for application in line with stakeholder intervention characteristic preferences and to establish joint expectations for implementation protocol.

## Results

**Figure 2.**
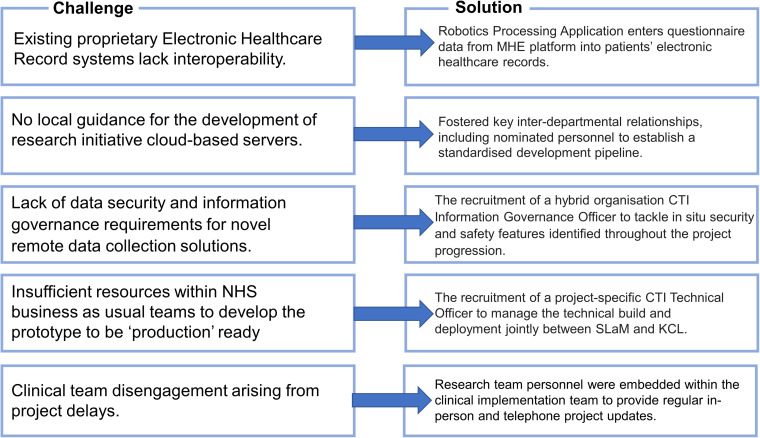
Summary of MHE translational challenges and solutions.

Below, we provide a narrative account of the MHE development and implementation efforts. Figure 2 provides a summary of the solutions resulting from our anticipated translational challenges.

### Data security and information governance

To identify and prevent privacy breaches, new ICT initiatives developed in the NHS environment for use with PII are required to undergo a data privacy impact assessment. Following an initial security review, the web application server hosting the MHE prototype failed to meet privacy protection standards. A replacement web application server within the Microsoft Azure cloud platform was identified as an appropriate host for the multi-agent system. Following subsequent Microsoft Azure information governance approval, funding was secured to provision two MHE-specific internal servers and to ensure appropriate data protection and system security features were built. Penetration testing on the agent code and data flow connectivity was outsourced to a SLaM-endorsed industry partner. All password-protected data repositories are located within the SLaM firewall, and a disaster recovery strategy has been developed for system restoration if a critical issue is encountered.

### myHealthE application profile refinements

**Figure 3.**
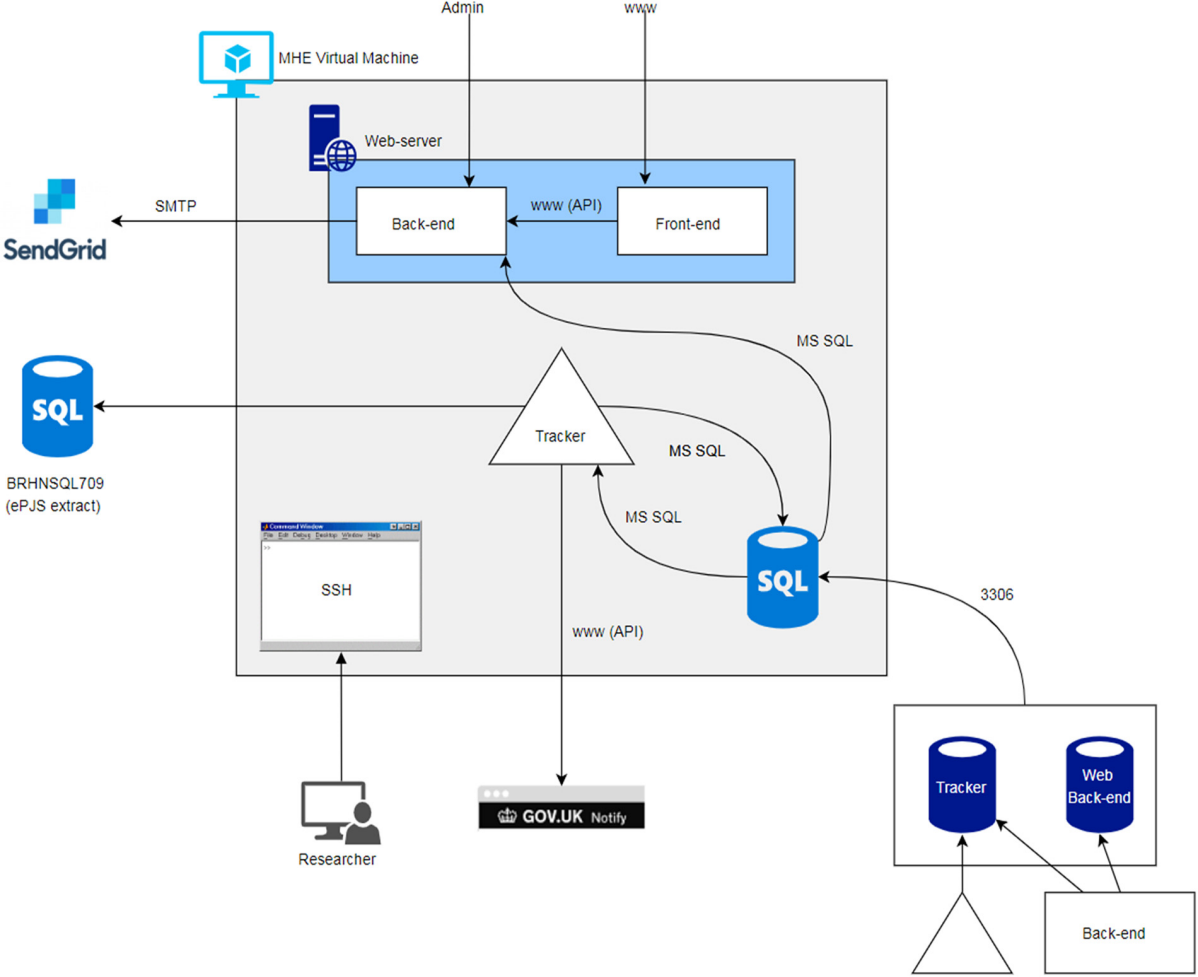
Internal MHE architecture.

Overall, the new MHE environment took 23 months to build. The internal Azure setup is provided in Figure 3.

The critical pieces are as follows:

– A web application frontend (patient/user-facing website), web application backend (content management system and database interface for the frontend), and tracker agent (Java-based ePJS interface, scheduler, and notification dispatcher);
– Core system components – web server (Apache, Inc. PHP, serves web frontend and backend), application database server (local MySQL instance, hosts backend content management system and logins, and SDQ/tracker data), and Azure server instance (CentOS Linux server, runs above components);
– External dependent services – Gov.UK notification service (service/tracker emails and SMS), Cloudflare network (traffic routing, TLS certificate), SendGrid mail service (password reset emails), and ePJS database (if new patients/SDQs need to be ingested).

To make information collected via MHE available for potential clinical use, we aspired to enter this data directly into SLaM's main electronic patient records system – ePJS. However, initial scoping revealed that ePJS does not provide an Application Processing Interface (API) to allow for data transfer. During development, the MHE team were approached by the Trust's clinical systems team with an opportunity to trial a novel data processing tool – NDL Automate Robotics Processing Application (RPA) software (https://www.ndl.co.uk/products/ndl-automate/), which would enable this. In brief, RPA runs visual basic scripts programmed to simulate data entry through the frontend of patients’ EHRs, i.e. logging into ePJS, entering patients’ unique identifiable Trust ID, selecting active episodes of care, and inputting data points. A 6-week proof of concept was undertaken in which the RPA process was trialled on both the test and live ePJS platforms, resulting in a successful data transfer for new SDQ-P data collected via MHE. To date, RPA software has been deployed successfully by several NHS Trusts to accelerate end-to-end data transmission, including The Princess Alexandra Hospital (https://www.ndl.co.uk/media/ybyn421 s/princess-alexandra-nhs-end-to-end-referrals-case-study.pdf), Mid Yorkshire Hospitals (https://www.ndl.co.uk/success-stories/digital-front-door-cypt-services/), Nottinghamshire Healthcare NHS Foundation Trust (https://www.ndl.co.uk/success-stories/nottinghamshire-healthcare-nhs-foundation-trust-02-23/), and Hertfordshire Community NHS Trust (https://www.ndl.co.uk/success-stories/digital-front-door-cypt-services/).

### Performance testing

The MHE team worked iteratively with the frontend application developer to design the MHE website, with development starting in May 2018 and lasting approximately 6 weeks. The patient-facing platform was informed by pre-development exploration with a convenience sample of 5 caregivers. Discussion topics mainly focused on the acceptability and practicability of a DHMS for supporting their child's treatment, relating to remote administration and completion of routine outcome measures. This included the platform's functions, access, and ease of use, as well as concerns around privacy and family burden, i.e. email and text message frequency.

The resulting prototype was thoroughly tested for technical glitches by the MHE team throughout development using dummy login credentials. Testing was first conducted on the high-performance and research cloud computing platform, *Rosalind*, which is hosted by KCL, and is co-funded by and delivered in partnership with KCL and the National Institute for Health Research. It was subsequently moved to a live MHE staging platform located behind the SLaM firewall, to enable full ecological testing, which included inputting new SDQ data, account registration verification, and website access via an extensive range of operating systems and internet-enabled devices.

### Development barriers

**Figure 4.**
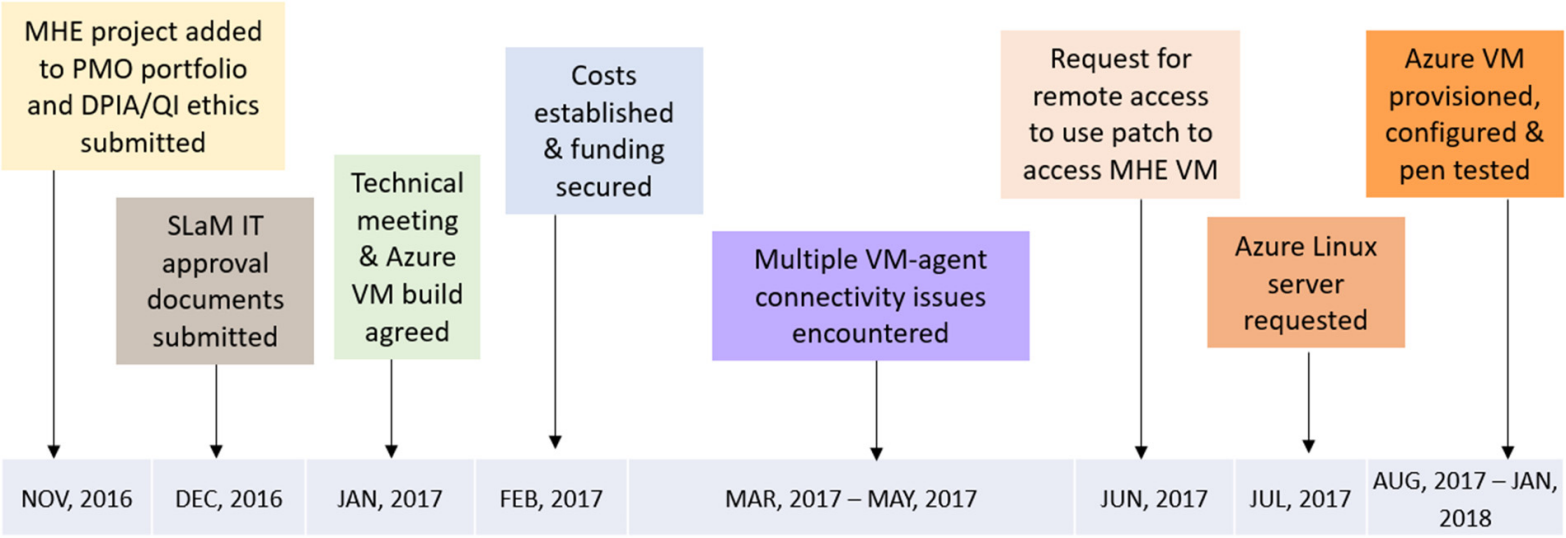
MHE development and implementation timeline.

At the time of development, no firm guidelines were in place for implementing new digital innovations into SLaM NHS infrastructure. Clarity regarding SLaM's product development procedures was obtained step-by-step with assistance from SLaM advisory groups, individuals on the Centre for Translation Informatics (CTI) operations board (a research partnership jointly led by SLaM and KCL aspiring to improve healthcare using digital innovations), Project Management Office, and SLaM and KCL Information Governance personnel. Accordingly, we spent a substantial amount of time becoming versed in the governance, logistical and technical resources, and funds required to implement and sustain projects within SLaM IT infrastructure as described in Figure 4. We worked closely with SLaM IT's operations department to achieve the resulting MHE technical build. However, due to the sensitive nature of the data being collected and processed by MHE, it was difficult to deliver on our time-sensitive grant-funded milestones owing to SLaM’s understandable need to manage business-as-usual and innovative projects simultaneously within their existing workforce. Therefore, despite having the in-house KCL technical skills and resources to identify, monitor, and resolve inevitable bugs, we were reliant on SLaM-dedicated technicians to make changes to firewalls, Content Delivery Networks, and ports to support its development and maintenance.

### myHealthE implementation

#### Baseline evaluation findings

We used CRIS to ascertain current levels of parent-reported SDQ collection for the participating service as well as all other child mental health services served by SLaM. Of the 28,382 CAMHS service user records surveyed, baseline SDQ-P was observed for approximately 40% (*n* = 11,212) of the sample, and of these cases, only 8% (*n* = 928) reported follow-up SDQ-P in the subsequent 6 months.^20^ These findings highlight the substantial disparity between the recommended use of repeated PROMs in line with National guidance^5^ and the current practice observed within the Trust. Thus, they demonstrate an area where the benefits of implementing MHE may surpass traditional pen-and-paper PROM collection methods, enabling a streamlined approach to the delivery, completion, and processing of PROMs. Considering the CFIR, this demonstrates the potential relative advantage of MHE over conventional approaches, which plays a crucial role in enhancing the chances of successful implementation.^56^

#### Orientation, stakeholder engagement, and system refinement

Site participation was agreed upon through professional links between the research team clinical lead and service managers. AM and JD initially presented the use case and project aims for MHE, including the findings from the baseline evaluation. Subsequently, AM attended routine clinical meetings at the participating service on a fortnightly basis to establish joint expectations and responsibilities for the rollout of MHE. Guided by the CFIR principles of inner settings readiness and individual characteristics, these meetings developed the team's appetite for implementation by providing an opportunity to communicate the available resources on hand to support implementation and develop the team's self-efficacy regarding their knowledge and understanding of MHE.^56^ Team visits were also used to provide real-time updates on product development. Delays in server development and application–server connectivity meant that this stage continued beyond the allocated 6-month period for an additional 12 months before the feasibility trial initiation. Professional links were maintained through email updates and clinic visits from AM, though visit frequency was reduced during this extension to enable more meaningful progress presentations.

Accordingly, stakeholder engagement activities commenced before a complete MHE prototype was available. Using an earlier prototype of the MHE platform, two user-testing sessions were undertaken with the clinical team (ranging from seven to 10 attendees) to collect stakeholder feedback. In these sessions, AM began by providing a dummy run-through of the MHE portal from a user perspective, which provided staff with an idea of the frequency and content of MHE communications caregivers, web-portal aesthetics, SDQ data entry requirements, and response output. After which, the staff were allowed to interact with the platform themselves. With regards to the CFIR, these activities served several purposes; notably, user-testing demonstrated the minimal complexity associated with MHE, from both the perspectives of the service users interacting with the system and the clinician's ability to retrieve data entered via the platform, which was unchanged from where they would usually view this data in the patients EHRs. It also brought to light the potential impact on cost and burden for the clinical team in terms of their workload due to timesaving associated with automated distribution and data entry.

Through transparent open-ended discussion, the staff raised initial questions and comments about procedural differences between automated data collection and the current distribution and collection of baseline and follow-up SDQ-P forms. Concerns centred on remote technology detracting from the therapeutic process through (a) limiting the opportunity to use SDQ-P responses completed in-session as a springboard to identify underlying concerns that may not be otherwise disclosed or not be possible for the clinician to identify by simply reviewing remotely completed SDQ-P results; (b) caregivers’ potential to misunderstand questions or misinterpret SDQ-P output which could be negatively fed back to the patient; and (c) creating a lack of caregiver motivation due to online completion being perceived as a tick box exercise. Following these discussions, the clinical team viewed the proposed introduction of the MHE system into routine care with overall positive regard. Suggested frontend design changes from these sessions included the use of different background images to reflect the diversity of service users, additional features on the graphics used to present questionnaire response data back to caregivers and text content, relating to platform security and how information collected via the platform is managed and used. Alterations to the platform were made on a retainer contract basis, meaning the platform appearance was adaptive to issues identified through comprehensive within-team and user testing.

We were unable to replicate the clinical staff user-testing sessions with caregivers, due to time issues with the final MHE build. However, the same process was applied to a convenience sample of caregivers (*n* = 3) on an individual basis who agreed to meet with AM following a clinical appointment to assess caregiver perspectives on the notion of using MHE to support their child's treatment. Features that were endorsed by caregivers centred on the clear layout of the website, trusted NHS branding, and the anticipated ease of completing routine outcome measures. Once a working prototype of the current MHE platform was available, security issues associated with the MHE NHS firewall-protected server configuration delays continued to impair our ability to invite patients and caregivers to experiment with the platform outside of the clinic to obtain ecologically valid feedback.

#### Preliminary MHE feasibility findings

Following final system changes, all caregivers of active Lewisham NDT patients were contacted by letter to inform them of potential changes to the way Lewisham CAMHS gather clinical information about their patients (i.e. electronic rather than paper questionnaires) and contact information should they have any questions about the initiative. We witnessed a high turnover of staff within the Lewisham NDT service during the protracted pre-implementation stages which was unrelated to the study, rendering new team members less familiar with the trial and its purpose. Therefore, we decided to perform the trial using a single-blind study design, which means that clinicians would be unaware as to whether their patients were receiving MHE monitoring or standard care to minimise any undue influence of clinician's behaviour, such as encouraging MHE use on user engagement. The Lewisham clinical lead was contacted 2 weeks before the trial start date to obtain final approvals.

A total of 196 families who had completed at least one SDQ-P were enrolled on the trial (MHE provided *n* = 98 and care as usual provided *n* = 98). A total of 70% of caregivers who received the MHE system filled out a minimum of one SDQ-P during this period compared to 8% of caregivers who continued with standard paper-based reporting. Over the period of 3 months, 87 follow-up SDQ-Ps were recorded via the platform. All except one caregiver, who registered on their personal MHE account, continued to complete an electronic SDQ-P. Integration of MHE was mainly positive except for one identified barrier, where caregiver contact details were only present in the expected area of ePJS for over half the patients enrolled to receive MHE. This meant that AM had to screen other structured or free text fields in ePJS to identify the necessary contact information needed to enrol a patient on the trial and enter it directly into the MHE backend to onboard the remaining patients. All data collected via MHE was successfully imported to the patient's EHRs using RPA, making this information immediately available to the patient's treating clinical team in the same way as paper-reported SDQ-P data.

## Discussion

This paper describes the implementation of state-of-the-art concepts in informatics research bearing immediate translational benefits to clinical care in mental health. We document the rationale and methodology supporting the development of the MHE system within modern NHS technical infrastructure and key barriers surmounted to successfully deploy MHE within clinical systems.

### Added value of MHE

PROMs offer enormous potential to improve the quality of mental health services.^57^ In MHE, we have designed and provisioned an online monitoring tool fully integrated with NHS electronic records to automate the collection of patients’ reported clinical information at pre-defined, repeated post-treatment time points and to facilitate associated patient communication electronically. While electronic systems have demonstrated considerable promise to facilitate PROM uptake,^58–60^ current examples of clinical value are more readily reported for physical health^34,59^ or, in cases where digital measurement-feedback systems have been implemented in child mental health services, do not interface directly with patient EHRs (https://patient-tracker.co.uk/cyp-camhs-iapt-outcome-measures/)^61^. While successful implementation of eHealth platforms into existing health records has been reported in adult health,^39^ to our knowledge, MHE is the first of its kind to achieve this in child mental health services in the UK.

Similarly, to other initiatives that have attempted to improve rates of measurement-based care in child mental health services, we observed levels of caregiver SDQ completion that were substantially higher than rates reported for current paper-based practices.^61^ These findings suggest that through automated patient communication and availability of patient-reported information, digital health platforms have the potential to tackle some barriers to routine outcome monitoring frequently reported in the literature,^24,25,62,63^ particularly the time-limiting step of documenting PROMS. As such, MHE may improve the transparency of care, by automating patient involvement in the process of collecting audit data and the efficiency and quality of care, by ensuring that clinicians are aware of their patient's progress. Moreover, given the rapid shift to remote service delivery brought about by the COVID-19 pandemic and the NHS's ambitious plan to digitalise the health sector over the next 10 years,^64^ paperless health monitoring innovations must outperform the current practice in a way that is safe and agreeable to patients and their healthcare providers.

This paper has highlighted how difficult implementation efforts are. Even with the best laid out plans, delays in other elements of the project combined with the need to complete the implementation cycle within a 24-month charity-funded budget time window had a ripple effect on on-site preparation. Regular in-person research visits to the clinic meant we were able to conduct thorough user-feedback activities with the clinical team, leading to iterative alternations to the MHE platform, which ultimately boosted the team's ownership of the product and increased their confidence and knowledge around its functionality, in keeping with CFIR recommendations.^56^ Notably, the technical barriers majorly influenced our ability to involve families in the design of the patient portal. This is problematic since patient portal engagement is strongly influenced by the patient's interest and capacity to use web-based portals.^65^ Early indications from our feasibility trial suggest that the current platform was acceptable to families; end-user follow-up will be conducted to assess the acceptability of this current platform and how it can be improved to increase MHE engagement rates.

### Sustainability and scalability challenges

In keeping with the intervention characteristics of the CFIR, trialability is central to long-term implementation success.^56^ As such, the positive impact on SDQ-P collection following the deployment of MHE led to an expression of interest from senior CAMHS management to scale up the systems use across all of SLaM child mental health services. Capacity issues within SLaM IT made it uncertain how long response times would be when faced with inevitable bugs and technical difficulties. CTI-dedicated personnel were hired to ease the burden of MHE development (and other CTI projects) placed on the digital services team who share research, development, and operational responsibilities, which notably helped communication between product developers and SLaM IT. However, continued collaboration between research and IT teams is needed to establish how this role can further streamline the development–test–production pipeline. For example, providing trained CTI staff with the authority and operational capability to action approved changes from SLaM's IT operations change acceptance process. Adjustments to firewalls, network, and port changes are examples of operations that can hinder development progress, which could have a considerable impact on service user engagement, and likely dampen clinical service enthusiasm for being an implementation trial site.

A key technical challenge will be maintaining MHE constituent component harmonisation once rolled out across the Trust, to ensure uninterrupted data flow functionality. Monitoring insights were set up by the research team and CTI throughout development. However, the responsibility for checking these reports for each of the system's critical components and performing system maintenance, i.e. software updates, will fall under the remit of SLaM IT once the system is added to the hospital's business-as-usual monitoring portfolio. Therefore, careful planning is needed to document and agree on these procedures ahead of a full-scale launch to ensure that adequate resources are available to support system amendments, advancements, and restoration promptly.

### Planned system expansion and application

Valuable findings from our feasibility trial highlighted ways the system could be improved. For example, the difficulties experienced extracting caregiver contact details from structured clinical records, which could be remedied by manualising caregiver contact information entry upon MHE registration, allowing MHE to search multiple EHRs locations by refining the SQL scripts responsible for this process, or using natural language processing methods to identify this information in free text records. Plans are in motion to expand the MHE framework to increase functionality and support routine care pathways, including referral tracking, information gathering, and signposting features, as well as the addition of condition-specific outcome measures and adult PROM questionnaires to facilitate mental health service delivery more broadly. MHE also offers a unique opportunity under a consent-based research ethics framework to support the collection of more experimental data, and to facilitate research recruitment and delivery. At present, funding has been secured to interface with other technologies capturing neuropsychological and accelerometer data (https://fundingawards.nihr.ac.uk/award/CS-2018-18-ST2-014) and identify patients eligible to use an online parenting intervention platform (https://fundingawards.nihr.ac.uk/award/RP-PG-0618-20003). MHE implementation has equipped us with the knowledge and technical support to build and deploy solutions in NHS-walled servers’ phase two development, which should be less time-consuming and allow more time for planned theoretically informed end-user testing.

## Conclusion

We provide a worked example of multi-agent platform development to improve patient-reported outcome collection using timely personalised communications to guide self-care outside the hospital environment, strengthen families’ sense of support, and increase their commitment to treatment. Such frameworks provide a cornerstone in the applicability of digital health outcome monitoring research, a relatively young field with large potential but few real-world applications in mental health clinical practice. By overcoming barriers to operating within NHS clinical systems, MHE can automate routine clinical data collection, which is infrequently reported in CAMHS. The system will ease clinician burden and provide a bridging connection with their patients using methods beyond the scope of current clinical practice.

## Acronym table

APPROaCh: Agent Platform for automating patient PROvided Clinical outcome feedback
CAMHS: Child and adolescent mental health services
CFIR: Consolidation Framework for Implementation Research
CRIS: Clinical Records Interactive Search
CTI: Centre for Translation Informatics
DHMS: Digital Health Monitoring System
EHR: Electronic Health Record
ePJS: electronic Patient Journey System
ePROM: electronic Patient-Reported Outcome Measures
IT: Information Technology
KCL: King's College London
MHE: myHealthE
NDT: Neuro-developmental Team
NHS: National Health Service
PII: Patient-Identifiable Information
PROM: Patient-Reported Outcome Measures
RPA: Robotics Processing Application
SDQ-P: Caregiver-reported Strengths and Difficulties Questionnaire
Slam FT: South London And Maudsley Foundation Trust
